# Structural analysis of bacterial metallothioneins involved in heavy metal detoxification

**DOI:** 10.6026/973206300222957

**Published:** 2026-05-31

**Authors:** Deepra Ghosh, Medha Kumari, Kriti Kumari, Bappa Biswas, Pritam Lenka, Sajalendu Ghosh

**Affiliations:** 1 Department of Statistics, Operations and Data Science, Fox School of Business, Temple University, Philadelphia, PA, 19122; 2 Department of Zoology, Dr. Shyama Prasad Mukherjee University, Ranchi, Jharkhand 834008, India; 3 ICMR-Regional Medical Research Centre, Bhubaneswar, Odisha 751023, India

**Keywords:** Metallothionein (MT), heavy metal detoxification, in silico structural analysis, protein homology modelling, microbial bioremediation, phylogenetic analysis

## Abstract

Microorganisms have developed many ways to circumvent heavy metal stress, mainly by synthesizing metallothionein-like proteins. Heavy metal pollution is a serious environmental concern because it lasts for a long time, is highly toxic and builds up in living organisms, posing risks to both ecosystems and human health. This microbial defense against heavy metal cytotoxicity can be proven to be a game-changer in heavy metal bioremediation if properly understood in terms of size, structure and biochemical nature. Hence, four bacterial metallothionein-related proteins, Smt, MymT and BmtA, were analysed. Data shows differences in size, stability and structure: larger proteins tended to be more stable with higher aliphatic indices, while smaller ones were more flexible and still preserved key metal-binding motifs. Thus, we show the structural and evolutionary features of these proteins to understand their roles in microbial metal detoxification and bioremediation.

## Background:

Within the last few decades, environmental pollution has increased significantly due to rapid industrialisation and human expansion [1, 2]. Among the leading contributors, heavy metals are of special concern since they are persistent, non-biodegradable pollutants that can accumulate in natural ecosystems [2, 3, 4]. Metals like Arsenic (As), Lead (Pb), Cadmium (Cd), Zinc (Zn), Mercury (Hg) are reportedly known for high-toxicity levels [4, 5]. These are predominantly introduced into soil and water systems through mining activities, industrial effluents, metallurgical processes, agricultural fertilisers and pesticide application in excess [6, 7]. Once released into the environment, these metals can persist indefinitely and bioaccumulate through ecosystems [8]. Heavy metal pollution is a major threat to both environmental and human health. Bioaccumulation of metals in aquatic ecosystems contributes to unsafe drinking and irrigation water, while also posing danger to aquatic fauna [9]. Similarly, accumulation of heavy metals in soils inhibits microbial activity and reduces soil fertility, thereby impacting agricultural productivity and eventually human health through the food chain [10]. Consequences involve a variety of health problems, including neurological disorders, kidney damage and respiratory complications [1, 1], where higher levels are associated with suboptimal biological performance. Exposure to harmful concentrations of heavy metals has emerged as a concerning topic, particularly relevant to the context of bioaccumulation and biomagnification [13]. Certain metals, such as Cu, Zn and Ni, are required in trace amounts for normal cellular physiology; however, increased levels become toxic and can alter cellular metabolism, affecting various pathways [14, 15]. At the cellular level, heavy metal-induced reactive oxygen species (ROS) production interferes with numerous biological processes, inhibits enzyme activity, damages cell membranes and alters protein and nucleic acid structures [16, 17, 18]. Microorganisms have developed mechanisms such as metal efflux systems, enzymatic transformation, extracellular immobilization and intracellular sequestration to combat heavy metal stress [19, 20, 21, 22, 23, 24, 25]. One of those important mechanisms includes microbial metallothionein (MT), cysteine-rich proteins that chelate metal ions via thiol groups to generate stable metal-thiolate complexes [26, 27]. These complexes function in maintaining heavy-metal homeostasis to reduce the harmful effects of metals in cells while ensuring heavy metal stress tolerance [28, 29]. The structural diversity and evolutionary relationships of many bacterial metallothionein proteins are important for metal detoxification [30, 31], but have not been studied extensively. The in silico approaches provided better resolution on protein structure and function and bioinformatics analysis detailed the protein architecture and phylogenetics more efficiently [32, 33]. However, the discovery of preliminary structural features associated with heavy-metal binding and detoxification can help further understand their potential role in microbial bioremediation. Therefore, it is of interest to comparatively study different bacterial metallothionein-like proteins to better understand the bacterial heavy-metal detoxification mechanisms.

## Materials and Methods:

## Retrieval of amino acid sequences:

The amino acid sequences of metallothionein-related proteins were retrieved from the NCBI protein database and analyzed using BLAST (Basic Local Alignment Search Tool) [34, 35]. Four representative proteins, SAM-MT, PEMT, MymT and BmtA, were selected. Homologous sequences showing ≥96% similarity were used for further comparative analysis.

## Physicochemical characterization:

The ProtParam tool available at the ExPASy server was employed to analyze the physicochemical properties of the selected metallothionein proteins [36]. This tool calculates amino acid composition, theoretical pI, extinction coefficient, instability index, aliphatic index and Grand Average of Hydropathy (GRAVY). These parameters played a crucial role in assessing the stability, hydrophobicity and structural features of each protein.

## Secondary structure prediction:

The secondary structure characteristics of the MT proteins were predicted using the Self-Optimised Prediction Method with Alignment (SOPMA) tool [37]. It predicts the secondary structure content (proportion in terms of alpha helices, extended strands (betas), beta turns and coils) in an amino acid sequence. Also, the percentage distribution of these features gave us information on the structural organization and stability potential of the proteins.

## Homology modeling and structural validation:

The tertiary (3D) structures of the selected metallothionein proteins were predicted via the SWISS-MODEL server [38]. Template coverage and sequence identity were used as guidance for aligning target sequences with suitable templates from the Protein Data Bank (PDB) and building structural models in the modeling process. The stereochemical quality of the predicted models was evaluated using Ramachandran plot analysis [39, 40], which examines the distribution of amino acid residues according to their backbone dihedral angles phi (ϕ) and psi (ψ). The proportions of residues located in favored, allowed and disallowed regions were used to assess the reliability and structural stability of the predicted protein models.

## Phylogenetic analysis:

Multiple sequence alignment identified the conserved regions, followed by a phylogenetic analysis, performed in MEGA-X to investigate the evolutionary relationship between the studied MT proteins. The phylogenetic tree was constructed using the Maximum Likelihood method [41, 42]. Bootstrap analysis with 1000 replicates was used to assess the reliability of the tree topology and to provide information on evolutionary divergence and clustering patterns of metallothionein proteins from different bacterial species.

## Statistical methodology:

**Figure 1:**
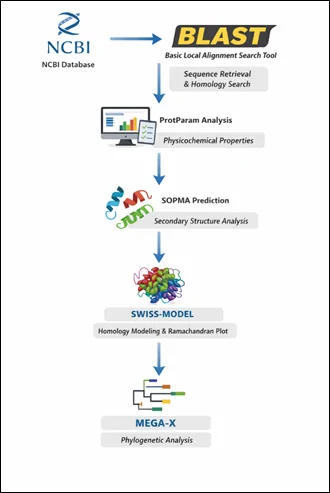
Overview of the workflow used for metallothionein protein analysis. Sequences were first retrieved from the NCBI database using BLAST. Their physicochemical properties were then analyzed with ProtParam, followed by secondary structure prediction using SOPMA. Three-dimensional structures were generated through SWISS-MODEL and evaluated using Ramachandran plots. Finally, phylogenetic relationships were examined using MEGA-X.

Physicochemical characterization of 17 protein accessions comprising SAM-MT (1-6), PEMT (7-13), MymT (14-16) and BmtA (17) was conducted by analyzing six primary parameters: Amino Acid count, Theoretical pI, Extinction Coefficient, Instability Index, Aliphatic Index and Grand Average of Hydropathy (GRAVY). To ensure statistical comparability across variables with disparate numerical scales, all data were subjected to Z-score normalization (see PDF) before analysis in R Studio v2025.05.1. Bivariate associations were evaluated using a Pearson correlation matrix, while multivariate grouping was performed through Hierarchical Cluster Analysis (HCA) utilising Euclidean distance and Ward's minimum variance method. To visualize the high-dimensional relationships and identify key drivers of variance, Principal Component Analysis (PCA) was executed using the prcomp function, with the first two principal components retained for biplot construction. Final data visualization and cluster partitioning were implemented using the tidyverse, corrplot and factoextra packages, with convex hulls applied to define the geometric boundaries of the four identified protein families within the PCA space. The following flowchart (Figure 1) summarizes the workflow of the current study.

## Results:

**Figure 2:**
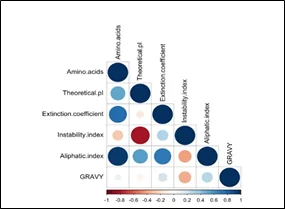
Correlation matrix of physicochemical properties of selected metallothionein proteins.

**Figure 3:**
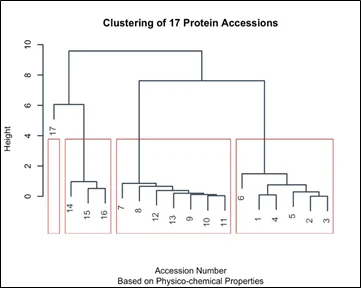
Dendrogram showing how the 17 metallothionein proteins cluster based on their physicochemical properties. Proteins with similar characteristics group together into distinct clusters (highlighted by red boxes), reflecting their related functions and properties.

**Figure 4:**
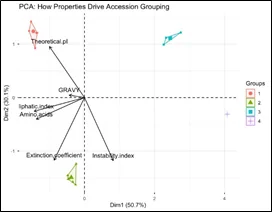
PCA biplot showing the distribution of metallothionein proteins and the contribution of physicochemical properties to their grouping. Arrows represent variable loadings, indicating how factors such as instability index, extinction coefficient and amino acid composition influence cluster separation.

**Figure 5:**
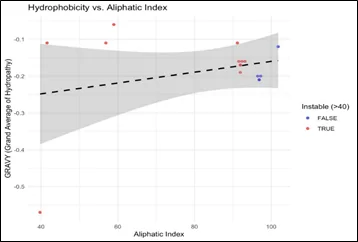
Relationship between GRAVY and aliphatic index of metallothionein proteins.

A total of seventeen MT protein sequences were retrieved by BLAST from the NCBI database with representative MT proteins as queries. Four protein groups were included: SAM-MT, PEMT, MymT and BmtA. Only sequences showing ≥96% similarity were selected. These proteins were obtained from bacterial taxa, including Pasteurella aerogenes, Pasteurella mairii, Acetobacter ascendens, Acetobacter pasteurianus, Mycobacterium avium complex and Comamonas sp (Table 1). Physicochemical properties predicted by the ExPASy ProtParam tool reflected molecular insights and stability of the studied proteins (Table 2). In contrast, MymT and BmtA were much smaller proteins with sizes of 45-80 residues, whilst SAM-MT and PEMT seemed to be larger, at around 240-260 amino acids. Accordingly, the molecular weights of the larger proteins ranged from 10,500 to 20,000 Da, while those of the smaller proteins ranged from 3,300 to 3,700 Da. The theoretical isoelectric point (pI) values also varied among the proteins. SAM-MT proteins showed pI values between 7 and 9, indicating neutral to slightly basic properties, whereas PEMT, MymT and BmtA exhibited slightly acidic pI values ranging from 5 to 6. Instability index analysis suggested that only SAM-MT proteins were stable, while the other proteins showed values above 40, indicating lower stability. In addition, larger proteins exhibited higher aliphatic indices (>90), suggesting greater thermostability than smaller proteins (<60). The plot (Figure 2) shows pairwise relationships among amino acid length, theoretical pI, extinction coefficient, instability index, aliphatic index and GRAVY. Blue circles indicate positive correlations, red circles indicate negative correlations and circle size reflects the strength of the correlation. The correlation matrix provides a high-resolution view of the dependencies between the six protein parameters. A robust positive correlation was observed between Amino Acid count (Length) and the Aliphatic Index, indicating that larger proteins in this dataset consistently possess a higher proportion of aliphatic side chains (alanine, valine, isoleucine and leucine), which is often a proxy for increased thermostability. Furthermore, the Amino Acid count showed a strong positive association with the Extinction Coefficient. This is expected as a higher number of residues generally increase the presence of aromatic amino acids (Tryptophan, Tyrosine and Cysteine) that contribute to molar absorptivity. Interestingly, the theoretical pI exhibited a strong negative correlation with the Instability Index, suggesting that the basic proteins in this specific set tend to be more structurally stable under physiological conditions than those with lower pI values. To evaluate the overall similarity between the 17 accessions, a hierarchical clustering analysis was performed using Euclidean distance and Ward's minimum variance method (Figure 3). The resulting dendrogram reveals a hierarchical structure that splits the accessions into four distinct functional clusters based on their shared physicochemical signatures. Primary Divergence: The most significant bifurcation in the dataset occurs at a height of approximately 10, where Accession 17 (Group 4) is isolated from all other samples. This indicates that Accession 17 possesses a unique physicochemical profile, likely characterized by its extreme outlier status in size or hydropathy, which distinguishes it from the rest of the cohort. Sub-clustering: The remaining 16 accessions are further partitioned into three groups: Group 1 (Accessions 14, 15, 16): Characterised by high internal similarity and moderate distance from the larger cluster. Group 2 (Accessions 7-13): A tightly knit group suggesting highly conserved physicochemical properties. Group 3 (Accessions 1-6): Shows more internal variance but remains distinct from the other major branches. PCA was utilised to reduce the dimensionality of the six numeric variables while retaining 80.8% of the total variance (PC1 = 50.7%, PC2 = 30.1%). The PCA biplot (Figure 4) confirms the cluster assignments and illustrates the "drivers" of protein grouping. PC1 Axis (Length & Stability): The first dimension is heavily influenced by Amino Acids, Aliphatic Index and Extinction Coefficient. Accessions plotted on the negative side of PC1 (Groups 1 and 2) are defined by larger sequences and higher thermostability. PC2 Axis (Isoelectric Point & Stability): The second dimension is primarily driven by Theoretical pI (positive loading) and the Instability Index (negative loading). Group 1 (Red): These accessions are characterised by high Theoretical pI and lower Instability Index. Group 2 (Green): These accessions align with higher Extinction Coefficients and Instability Indices. Accession 17 (Blue): Occupies a remote position on the far positive end of PC1, reinforcing its status as an outlier with the lowest values for length and aliphatic content in the dataset. A slight positive trend was observed, with most proteins showing similar hydrophobicity. Notably, some proteins remain stable at higher aliphatic index values (~95-100), suggesting a possible stabilising effect of aliphatic content. The analysis of GRAVY (Grand Average of Hydropathy) scores against the Aliphatic Index (Figure 5) shows a general upward trend, modelled by a linear regression: (see PDF)

**Figure 6:**
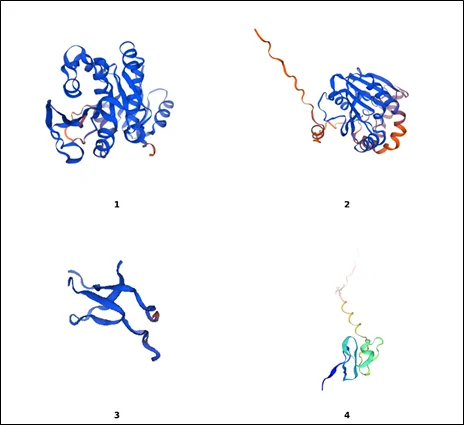
Predicted three-dimensional (3D) structures of four metallothionein-related proteins generated using SWISS-MODEL: (1) SAM-dependent methyltransferase, (2) Phosphatidylethanolamine N-methyltransferase, (3) MymT and (4) BmtA. Models were built using PDB templates with 45-75% sequence identity, showing good quality (GMQE: 0.60-0.78; QMEAN: -1.5 to -2.8) and typical secondary structural elements including α-helices, β-sheets and loops.

**Figure 7:**
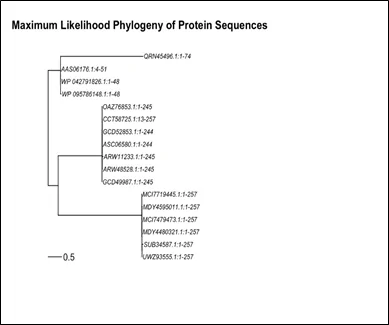
Ramachandran plots of the four SWISS-MODEL-generated metallothionein protein models showing residue distribution across ϕ-ψconformational space. Over 90% of residues fall within the most favoured regions, with few in additionally allowed and negligible in disallowed regions, indicating high stereochemical quality and reliability of the models.

**Figure 8:**
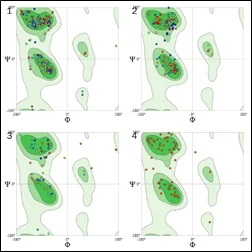
Phylogenetic tree showing the evolutionary relationships among metallothionein and related methyltransferase proteins from selected bacterial species. The proteins cluster into distinct groups, reflecting their functional similarity and shared evolutionary origin.

While most proteins fall within a narrow hydropathy range (-0.1 to -0.2), a significant number of accessions exceed the instability threshold of 40. However, the scatter plot reveals a critical "stability pocket" at the high end of the Aliphatic Index (approx. 95-100), where several accessions remain stable (purple points) despite their hydrophobicity. This suggests that high aliphatic content may be the primary stabilising factor for these specific proteins, potentially protecting them from denaturation. Secondary structure prediction of the selected metallothionein proteins was performed using the SOPMA (Self-Optimised Prediction Method with Alignment) tool. The analysis revealed clear differences in the distribution of structural elements among the four protein groups. PEMT had the highest content of α-helices, 36% to 47%, suggesting a stable and compact structural status. Stable folding is also indicated by the high α-helix content of SAM-MT proteins, ranging from 34% to 39% in the absence of ligands. Conversely, MymT proteins showed a significantly lower α-helix content (about 2%), while they were characterised by high levels of β-strands (41-42%) and random coils (56%). This composition implies a higher degree of structural flexibility. On the contrary, BmtA proteins exhibited moderate α-helical content at ~33.78%, combined with a considerable fraction of random coils at 60.81%, demonstrating partial flexibility. In conclusion, SAM-MT, PEMT and BmtA proteins were rich in α-helix structure, while MymT proteins were composed of more β-strands and random coils (Table 3).Sequence analysis further showed the presence of conserved cysteine residues typical of metallothionein. Their cysteine-rich domains facilitate the formation of metal-thiolate clusters and allow binding to heavy metals, including Cd^2+^, Zn^2+^ and Hg^2+^. The summary of the total proteins and cysteine residues identified is presented in Table 4, as these amino acids are crucial for metal ion binding and detoxification Table 3. Predicted secondary structure of metallothionein proteins using SOPMA, showing the proportions of α-helix,β-strand and random coil. Most proteins contain a mix of helices and coils, while MymT proteins are richer inβ-strands and coils. The three-dimensional (3D) structures of representative metallothionein proteins were predicted using homology modelling with the SWISS-MODEL server. Suitable templates were selected from the Protein Data Bank (PDB) based on sequence similarity and alignment coverage, with sequence identities ranging from 45-75%, ensuring reliable model construction. SWISS-MODEL provided Global Model Quality Estimation (GMQE) and Qualitative Model Energy Analysis (QMEAN) scores to assess model quality. Data were defined as having acceptable structural quality for further analysis, with GMQE scores ranging from 0.60 to 0.78 and QMEAN scores between -1.5 and -2.8. Predicted models contained structural elements consistent with secondary structure predictions incorporating α-helices, β-sheets and connecting loop regions. Further, the stereochemical quality was assessed using the Ramachandran plot, which evaluated the distribution of amino acids according to their phi (Φ) and psi ( Ψ) dihedral angles. More than 90% of residues fell into the most favoured regions and a small fraction into additionally allowed regions, suggesting excellent model reliability. The predicted tertiary structures and their corresponding Ramachandran plots are shown in Figure 6, Figure 7. The phylogenetic tree arranged the sequences according to their respective functional annotations, spanning PEMT, MymT, SAM-MT and BmtA. Proteins within the same functional category were clustered together, indicating high sequence similarity among phylogenetically related bacterial taxa. The clustering pattern reflected the evolutionary relationships among species across multiple bacterial genera, whilst retaining functionally conserved domains for metal binding (Figure 8).

## Discussion:

Contamination by heavy metals remains a serious environmental problem due to their non-biodegradability, toxic nature and potential bioaccumulation of various metal ions in natural environments [4]. Microorganisms that inhabit anthropogenically damaged sites possess several mechanisms to alleviate metal stress, such as efflux systems, enzymes capable of metal transformation, intracellular storage processes and the specific binding of metal ions to specialised proteins with high affinity [19,21,23]. Among these, MTs play a key role in balancing the intracellular metal homeostasis, which is accomplished by the binding of excess metal ions via cysteine-rich domains, leading to the formation of stable metal-thiolate complexes. Subsequent analysis of the physicochemical properties also showed significant variation in size, stability and hydrophobicity among MT-related proteins. This occurs in SAM-MT and PEMT, where the larger proteins are shown to be more stable regardless of their environment, as suggested by significantly higher aliphatic indices, implying greater content of aliphatic side chains. Aliphatic index plays an important role as a major factor of protein thermostability and structural stability [43]. In contrast, both MymT and BmtA were more unstable and had lower aliphatic indices, which indicates that they are more flexible compared to the larger proteins. The multivariate analysis of the 17 protein accessions untangled four distinct groups, out of which three matched exactly with their biological classification: SAM-MT (Accessions 1-6), PEMT (Accessions 7-13) and MymT (Accessions 14-16). BmtA kicked up as an outlier at Accession 17.

The cumulative contributions of all the protein families resulting from hierarchical clustering and PCA accounted for 80.8% of total variance, highlighting that these protein families have distinct physicochemical signatures. The SAM-MT and PEMT groups are situated in the negative side of PC1, showing positive correlation with amino acid number, aliphatic index and extinction coefficient, which indicates that these families are structurally more stable and thermally more robust than the others. BmtA, on the other hand, is a prominent outlier in the lower right of the PCA plot (Figure 4), revealing a specific arrangement with its compactness and reduced aliphatic content compared to those of methyltransferase clusters. The correlation analysis illustrates the dissimilarity among these groups and shows that protein length is positively correlated with the aliphatic index. This "length-stability" axis indicates that the longer sequences in the SAM-MT and PEMT groups have a greater fraction of aliphatic (hydrophobic) side chains, which are present to preserve stability. The second PC (PC2), reflecting a "charge-stability" trade-off with theoretical isoelectric point (pI) and the instability index, negatively correlated, better separates the basic MymT sequences from other clusters. In addition, the linear relationship between GRAVY and aliphatic index shows that while thermal stability increases with hydrophobicity, there is a so-called "stability pocket" for proteins rich in aliphatic groups. As a result, most accessions score under the 40-stability threshold; the SAM-MT and PEMT families have an ideal side chain packing to relieve possible structural disorder. Hydropathy analysis showed that most of the calculated GRAVY values were negative, suggesting that the analysed proteins were hydrophilic and are therefore suitable for intracellular aqueous environments. It is also noteworthy that conserved cysteine residues are representative of their involvement in metal binding. The characteristic feature of MTs is their cysteine-rich regions that allow for metal-thiolate clusters centred on heavy metals like Cd^2+^, Zn^2+^ and Hg^2+^ to bind [24]. In this way, MTs reduce the concentration of free metal ions and protect macromolecules in cells against metal-induced oxidative damage. Differences between the proteins were observed in secondary structure prediction. SAM-MT and PEMT had a higher α-helical composition, which may contribute to a relatively stable structural framework that may allow more efficient metal coordination [44]. Compared to the MymT proteins, there were significantly higher proportions ofβ-strands and random coils, thus indicating a more flexible structure that could adopt one or another stable conformation depending on which metal ions are being sequestered. The predicted folding topologies show good stereochemical quality, supported by the analysis of the Ramachandran plot. Most amino acid residues were in the favoured region, indicating that these structural conformations are reliable (Figure 5). The correct disposition of cysteine residues is required for the effective coordination of metals and stabilisation of metallothionein (MT) complexes [25, 45, 46]. Phylogenetic comparison showed that the proteins analysed were clustered according to their functional groups. Despite the diverse origins of these proteins from different bacterial taxa, they presented structural features associated with metal-binding activity that were conserved. This conservation suggests that MTs have effectively evolved while still retaining their fundamental functions in metal detoxification and cellular metal homeostasis [47, 27]. This work offers an overview of the physicochemical, structural and phylogenetic properties of bacterial MTs, their diversity and potential functional roles. It can also give an indication of their possible role in metal detoxification and bioremediation by microbes.

## Figures and Tables

**Table 1:** Metallothionein-related protein sequences retrieved from the NCBI database using BLAST. Seventeen proteins were selected based on ≥96% sequence similarity and grouped into four categories: SAM-MT, PEMT, MymT and BmtA. The table lists accession numbers, source bacterial species and protein types used for subsequent analyses.

**Sl. No.**	**Accession number**	**Bacterial species**	**Protein type**
1	MDY4480321.1	Pasteurella aerogenes	SAM-dependent methyltransferase (SAM-MT)
2	MCI7719445.1		
3	MCI7479473.1		
4	UWZ93555.1		
5	MDY4595011.1		
6	SUB34587.1	Pasteurella mairii	
7	ARW11233.1	Acetobacter ascendens	Phosphatidylethanolamine N-methyltransferase (PEMT)
8	OAZ76853.1	Acetobacter pasteurianus	
9	GCD52853.1		
10	GCD49987.1		
11	CCT58725.1		
12	ASC06580.1		
13	ARW48528.1		
14	AAS06176.1	Mycobacterium avium subsp. paratuberculosis	Mycobacterial metallothionein (MymT)
15	WP_042791826.1	Mycobacterium avium complex	
16	WP_095786148.1	Mycobacterium avium	
17	QRN45496.1	Comamonas sp.	Borrelia metal transporter A (BmtA)

**Table 2:** Physicochemical properties of selected metallothionein proteins predicted using ExPASy ProtParam, including amino acid length, pI, extinction coefficient, instability index, aliphatic index and GRAVY.

**Accession no.**	**Amino acids**	**Theoretical pI**	**Extinction coefficient**	**Instability index**	**Aliphatic index**	**GRAVY**
MDY4480321.1	257	7.78	10555	33.37	96.85	-0.207
MCI7719445.1	257	8.36	10555	33.67	96.85	-0.207
MCI7479473.1	257	8.36	10555	33.7	96.85	-0.207
UWZ93555.1	257	7.78	10555	33.37	97.24	-0.197
MDY4595011.1	257	8.35	12045	34.22	96.46	-0.196
SUB34587.1	257	7.16	10555	33.73	101.79	-0.121
ARW11233.1	245	5.51	18450	51.4	91.18	-0.109
OAZ76853.1	245	5.7	16640	49.12	91.96	-0.165
GCD52853.1	244	5.71	19940	48.78	91.93	-0.173
GCD49987.1	245	5.7	19940	48.78	92.37	-0.164
CCT58725.1	245	5.71	19940	48.9	93.14	-0.156
ASC06580.1	244	5.55	19940	48.75	91.93	-0.186
ARW48528.1	245	5.89	19940	49.06	91.55	-0.162
AAS06176.1	48	5.46	3355	41.56	41.56	-0.108
WP_042791826.1	48	5.46	3355	41.56	56.88	-0.108
WP_095786148.1	48	5.46	3355	43.32	58.96	-0.062
QRN45496.1	74	5.44	3605	60.35	39.73	-0.566

**Table 3:** Predicted secondary structure of metallothionein proteins using SOPMA, showing the proportions of α-helix,β-strand and random coil. Most proteins contain a mix of helices and coils, while MymT proteins are richer inβ-strands and coils.

**Accession no.**	**α-Helix (%)**	**β-Strand (%)**	**Random coil (%)**
MDY4480321.1	37.35	16.73	45.91
MCI7719445.1	36.58	17.9	45.53
MCI7479473.1	38.91	18.29	42.8
UWZ93555.1	38.52	18.29	43.19
MDY4595011.1	34.24	18.29	47.47
SUB34587.1	38.91	18.68	42.41
ARW11233.1	46.53	13.88	39.59
OAZ76853.1	37.96	13.88	48.16
GCD52853.1	38.93	13.93	47.13
GCD49987.1	40.41	13.06	46.53
CCT58725.1	38.37	13.47	48.16
ASC06580.1	40.16	13.3	45.9
ARW48528.1	38.37	14.29	47.35
AAS06176.1	2.08	41.67	56.25
WP_042791826.1	2.08	41.67	56.25
WP_095786148.1	2.08	41.67	56.25
QRN45496.1	33.78	5.41	60.81

**Table 4:** : Distribution of conserved cysteine residues in selected metallothionein proteins, indicating their roles in metal binding, structural support and metal sequestration across different protein types.

**Protein**	**Accession no.**	**Protein length (aa)**	**Number of cysteine residues**	**Predicted Role**
SAM-MT	MDY4480321.1	257	6	Potential metal-binding residues
PEMT	ARW11233.1	245	5	Structural stability and metal coordination
MymT	AAS06176.1	48	7	Metal-thiolate cluster formation
BmtA	QRN45496.1	74	8	Heavy metal sequestration
